# Complete chloroplast genome sequence of *Annamocarya sinensis* (Juglandaceae), an endangered perennial plant

**DOI:** 10.1080/23802359.2019.1681919

**Published:** 2019-10-30

**Authors:** Yancai Shi, Na Duan, Bingbing Liu

**Affiliations:** a Institute of Loess Plateau, Shanxi University, Taiyuan, Shanxi, China;; b Guangxi Institute of Botany, Guangxi Zhuang Autonomous Region, Chinese Academy of Sciences, Guilin, China

**Keywords:** *Annamocarya*, chloroplast genome, phylogenetic analysis

## Abstract

*Annamocarya sinensis* (Juglandaceae) is a tertiary relict tree restricted to southeastern China and northern Vietnam. Here, we first report and characterize its complete chloroplast genome based on Illumina paired-end sequencing data. The complete plastid genome was 160,065 bp, which contained inverted repeats (IR) of 25,786 bp separated by a large single-copy (LSC) and a small single copy (SSC) of 89,759 bp and 18,734 bp, respectively. The cpDNA contains 130 genes, comprising 85 protein-coding genes, 36 tRNA genes, 8 rRNA genes, and one processed pseudogene. The overall GC content of the plastome is 36.3%. The phylogenetic analysis of 18 selected chloroplast genomes demonstrated that *A. sinensis* was close to the species *Carya kweichowensis*.

*Annamocarya sinensis* (Dode) Leroy, a tertiary relict endangered tree, is the only species of the genus *Annamocarya* belong to family Juglandaceae. Its naturally distributes in the southwestern China and northern Vietnam bordering China (Zhang et al. 2013). As a tertiary relic, it is an important species in the phylogenomic studies of Juglandaceae. Due to its reduced and limited number of individuals, *A. sinensis* has been treated as critically endangered species in China and has been registered on the China Species Red List (Fu 1992). It is thus urgent to take effective measures to conserve this endangered endemic species. Herein, we first report and characterize its complete plastome based on Illumina paired-end sequencing data, which will contribute to the further studies on its genetic research and resource utilization. The annotated cp genome of *A. sinensis* has been deposited into GenBank with the accession number MN473449.

In this study, *A. sinensis* was sampled from Guangxi Zhuang Autonomous Region of China, located at 110°12′54″ E, 24°17′23″ N. A voucher specimen (Y.-C. Shi et al. H046) was deposited in the Guangxi Key Laboratory of Plant Conservation and Restoration Ecology in Karst Terrain, Guangxi Institute of Botany, Guangxi Zhuang Autonomous Region and Chinese Academy of Sciences, Guilin, China. The experiment procedure is as reported in Zhang et al. (2019). Around 2 Gb clean data were used for the cp genome de novo assembly by the program NOVOPlasty (Dierckxsens et al. 2017) and direct-viewing in Geneious R11 (Biomatters Ltd., Auckland, New Zealand). Annotation was performed with the program Plann (Huang and Cronk 2015) and Sequin (http://www.ncbi.nlm.nih.gov/).

The chloroplast genome of *A. sinensis* is a typical quadripartite structure with a length of 160,065 bp, which contained inverted repeats (IR) of 25,786 bp separated by a large single-copy (LSC) and a small single-copy (SSC) of 89,759 bp and 18,734 bp, respectively. The cpDNA contains 130 genes, comprising 85 protein-coding genes, 36 tRNA genes, 8 rRNA genes, and 1 processed pseudogene. Among the annotated genes, 14 of them contain one intron (*atp*F, *ndh*A, *ndh*B, *rps*16, *rpoC*1, *pet*B, *pet*D, *rpl*16, *rpl*2, *trn*A-UGC, *trn*I-GAU, *trn*L-UAA, *trn*S-CGA, and *trn*V-UAC), and three genes (*clp*P, *rps*12 and *ycf*3) contain two introns. The overall GC content of the plastome is 36.3%.

To identify the phylogenetic position of *A. sinensis*, phylogenetic analysis was conducted. A neighbour-joining (NJ) tree with 1000 bootstrap replicates was inferred using MEGA version 7 (Kumar et al. 2016) from alignments created by the MAFFT (Katoh and Standley 2013) using plastid genomes of 17 species. It showed the position of *A. sinensis* was close to the species *Carya kweichowensis* (Figure 1). Our findings can be further used for population genomic and phylogenomic studies of Juglandaceae. It will also provide fundamental data for the conservation, utilisation and management of this endangered endemic species.

**Figure 1.**
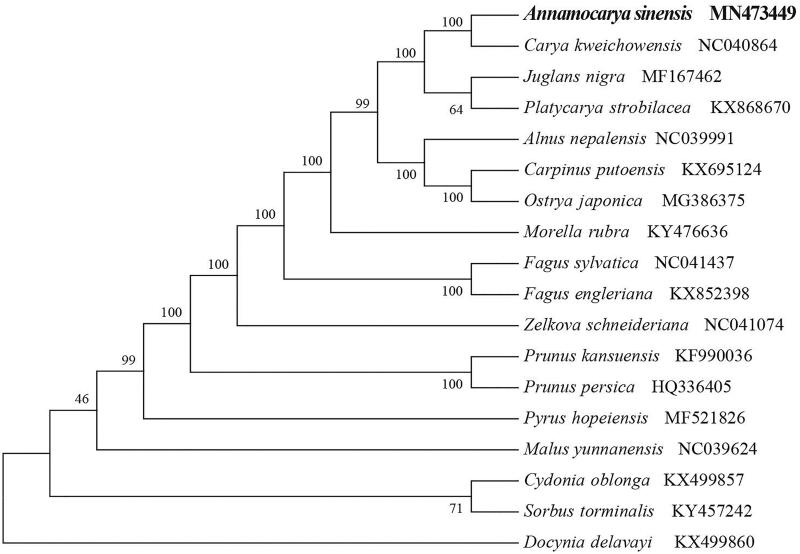
NJ phylogenetic tree of *A. sinensis* with 17 species was constructed by chloroplast plastome sequences. Numbers on the nodes are bootstrap values from 1000 replicates. *Docynia delavayi* was selected as outgroups.
